# Supplementary material for: Spatial production or sustainable development? An empirical research on the urbanization of less-developed regions based on the case of Hexi Corridor in China
Source: PLoS One. 2020 Jul 9;15(7):e0235351. doi: 10.1371/journal.pone.0235351 (PMC7347202; doi:10.1371/journal.pone.0235351)
Supplement: S1 File — (DOCX) [file pone.0235351.s002.docx]

Data Availability Statement

All relevant data are included in the Supporting Information files, which can be obtained by querying the relevant Gansu Development Yearbook. The vector data and the LULC data in the Hexi Corridor can be obtained freely from the Resource and Environment Data Cloud Platform (http://www.resdc.cn/) of Chinese Academy of Sciences. You need render your own map to display the data.
