# Supplementary material for: Spatial production or sustainable development? An empirical research on the urbanization of less-developed regions based on the case of Hexi Corridor in China
Source: PLoS One. 2020 Jul 9;15(7):e0235351. doi: 10.1371/journal.pone.0235351 (PMC7347202; doi:10.1371/journal.pone.0235351)
Supplement: Attachment — Submitted filename: Correction lists for PONE-D-20-09509.docx [file pone.0235351.s003.docx]

**Correction lists for PONE-D-20-09509**

Dear Samantha Russell:

Thank you very much for reviewing our manuscript of PONE-D-20-09509. This manuscript has been revised according to your instructions. Moreover, we have carefully improved the manuscript and the responses to the queries are listed below (All of corrections in text have been marked in color).

Best regards!

Dr. Zhi B Zhang

April 20, 2020

1. Please describe how the different analyses were conducted, the data used for each analysis, and the contribution of each analysis to your findings.

**Response:** Capital, power and class as the main driving forces of spatial production have played an important role in the urbanization process. In order to analyze the relationship between spatial production and urbanization, we used Ye Chao and Yang Zhen's research methods to construct a simple indicator system. We selected fixed asset investment and real estate investment to represent capital, per capita fiscal expenditure represents power, and urban-rural income ratio represents Class, urbanization rate is used to measure the level of urbanization (Ye, Yang).

In order to explain the development and evolution of the urbanization and spatial production of the Hexi Corridor, this article analyzes from the two dimensions of time and space. In terms of time, using the statistical data of FAI, PCFE, REI, UIR and urbanization rate of Gansu Yearbook (2001-2009) and Gansu Development Yearbook (2010-2018), it is characterized by Excel statistical charts, and at the same time, it combs the country and local The policy of the same period. This analysis helps to clarify the main driving force and role of urbanization in underdeveloped areas under multiple factors. This is of great significance to the basic characteristics of the spatial production of the asphalt Hexi Corridor and the role of different driving factors in the process of urbanization. In the social and spatial dimensions, we used the GIS method to visualize the spatial production process, and analyzed the spatial process and dynamics of the urbanization of the Hexi Corridor from the county level. The results laid the foundation for identifying urbanization development models in different regions and transforming governance strategies.

In order to further illustrate the impact of urbanization or space production on sustainable development, we mainly explain from two aspects. In terms of resources, use local government data (from the Water Resources Bulletin) to explain the dynamics and characteristics of changes in water use during urbanization; at the same time, use the RS method to interpret land use resource data in the Hexi Corridor to further illustrate the impact of urbanization development in less developed areas The impact of land resources. The conclusion of the analysis fully confirms the remodeling effect of urbanization on natural spaces. In terms of environmental protection, the destruction of the ecological environment of Qilian Mountains in China was selected as a typical case, and the mode of action of the main body of ecological destruction in the process of space production was analyzed. The results are of great significance for promoting the transformation of urbanization mode and spatial governance decision-making in less-developed regions.

2. Provide a sample size, sampling strategy, and describe questionnaire content for the social survey conducted.

**Response:** The social survey in the article is inaccurate. When analyzing the spatial and temporal evolution characteristics of spatial production and urbanization, we mainly use social and economic statistical data, and visualize with the help of Excel statistical analysis function and GIS method, the purpose is to reveal the changing process of different scales and changing characteristics.

3. Please describe how GIS and RS analysis was carried out.

**Response:** GIS spatial analysis: ①Data preparation: taking 20 counties (districts) in the Hexi Corridor as rows, four time nodes in 2000, 2005, 2010, and 2017 as columns, and the cells record annual FAI, PCFE, REI, UIR, and urbanization rate The numerical values constitute a two-dimensional spatio-temporal variable table; according to the vector and raster data of the national basic geographic information database, map data of the Hexi Corridor area is generated. ②Load data: Use Arcmap tool in ArcGIS to load map data and associate it with spatio-temporal data. ③Data Rendering: Render the unified graph symbols on the surface data, and then classify the spatio-temporal data. ④Map drawing: set the map size, add legend, scale, drawing information, and output the map.

RS analysis: ①Obtain satellite imagery from 2000, 2005, 2010 and 2015 from the resource and environmental data cloud platform of the Chinese Academy of Sciences to extract land cover information in four periods for interpretation. ②Use ArcGIS software to vectorize the interpretation map, and calculate various land areas according to various land attribute tables. ③Refer to the GIS method to make maps of various land area changes in the Hexi Corridor in different periods, and analyze the impact of urbanization on land resource utilization.

4. Describe in detail the literature form which theories and frameworks are drawn, and how they inform the methods.

**Response:** According to social-spatial dialectics, political power, economic capital and social class are the three important components of space production (Ye et al., 2017). Space production theory is concerned with the relationship between society and space. The traditional pattern of urbanization, dominated by government power, with large-scale expansion of space and capital, is almost at odds with the goal of sustainable development (Ye et al., 2017), this social relationship reshapes the process of urbanization from rural space to urban space. However, spatial production-led urbanization is changing human-land relationships as well as socio-cultural spaces in less developed regions (Fu et al., 2018). Urban-rural disparities have increased, spatial injustices have become more frequent, resource overuse and ecological damage have seriously affected regional sustainable development (Fang et al., 2019). The harmonious development of population, economy, resources and environment is the foundation of sustainable development (Tian and Sun, 2018). It is an inevitable choice for sustainable development to study the process and mechanism of spatial production and urbanization, and to explore the urbanization mode in harmony with ecological environment. On the basis of the above-mentioned theories, this paper puts forward a new conceptual framework by using the existing theoretical framework for reference. In describing the characteristics of spatial production and urbanization, we use the statistical analysis method. When analyzing the evolution process of spatial production and urbanization, we use GIS analysis method. The RS analysis method is used to explain the impact of urbanization or space production on the natural environment.
5. Please describe what changes you made to the manuscript and on what pages.

**Response:** The summary, the part two (2.3), and the part 3 (3.3) have been revised with emphasis on pages such as P1, P2, P3, P4, P9, P10, etc.  All of corrections in text have been marked in color.
